# Elite Athletes and COVID-19 Lockdown: Future Health Concerns for an Entire Sector

**DOI:** 10.3390/jfmk5020030

**Published:** 2020-05-07

**Authors:** Antonio Paoli, Giuseppe Musumeci

**Affiliations:** 1 Department of Biomedical sciences, University of Padua, 35131 Padova, Italy; 2 Human Inspired Technology Research Center, University of Padua, 35131 Padova, Italy; 3 Research Center for High Performance Sport, UCAM, Catholic University of Murcia, 30107 Murcia, Spain; 4 Department of Biomedical and Biotechnological Sciences, Human, Histology and Movement Science Section, University of Catania, Via S. Sofia n°87, 95123 Catania, Italy; 5 Research Center on Motor Activities (CRAM), University of Catania, Via S. Sofia n°97, 95123 Catania, Italy; 6 Department of Biology, Sbarro Institute for Cancer Research and Molecular Medicine, College of Science and Technology, Temple University, Philadelphia, PA 19122, USA

**Keywords:** COVID-19, prevention, physical activity, health, elite athletes

## Abstract

In this editorial, we focused our attention on elite athletes during the COVID-19 lockdown. A high level of physical fitness is required by elite athletes irrespective of the specific type of sport. Generally speaking, elite athletes avoid long periods of rest during and at the end of the competitive season. Normally, elite athletes stop training or reduce training volume and intensity for a period that ranges from two weeks to a maximum of four weeks.

## Introduction

The recent pandemic of COVID-19 forced elite athletes of different sports (basketball, football, soccer, track and field, swimming, etc.) to an unexpected stop not only of competitions but also of training sessions that, in some countries (Italy, Spain, etc.), will reach at least eight weeks. At the moment, in many countries, the proposal to re-start training programs in May and competitions in June, but a question arises: is one month of training enough to re-create the previous physical condition after two months of a complete stop? For some, on the surface, this might seem a minor issue during these perilous times, but for hundreds of thousands of elite athletes around the world this is not the case.

It is well known that long term detraining, as for this COVID-19 forced stop, leads to a marked decline in maximal oxygen consumption (VO_2max_), the loss of the recently acquired gains in term of endurance capacity, and, more importantly, to a loss of muscle strength and mass [1]. Even though the reduction of aerobic capacity impairs general performance, this would not be a serious issue considering that all athletes will be in (more or less) the same condition when they are allowed to train again. What should raise serious doubts about an early re-start of the competitive seasons is muscular–tendon condition. In eight weeks of detraining, athletes will lose muscle mass, muscle strength and power with a decrease of electromyographic activity (EMG), reflecting reduced muscle activation [2]. Such a reduction in skeletal muscle activity could significantly increase the risk of injuries both in non-contact and contact sports like soccer [3]. More importantly, after a sudden detraining period (like this for the COVID-19 pandemic) tendon structure and properties show an alteration of their tissue structural organization and of their mechanical properties [4]; the final result is an impairment of the normal tendon reaction to load application (Table 1).

Furthermore, inactivity contributes to the drastically decreased lubrication and nutrition (hyaluronic acid and lubricin) of joint cartilage, resulting in possible degeneration and in an imbalance in the maintenance and preservation of all the structures of a joint (cartilage, ligaments, synovium) [5,6,7,8]. Physical exercise represents a good strategy to preserve function, decrease pain and fatigue, and increase muscle strength and joint flexibility as well as joint tribology [5,6,7,8,9].

A similar testimony, which denotes the problematic treated in this editorial, regarding the forced stop of athletes, was the National Football League (NFL) Lockout during 11 March to 25 July 2011. During this time professional football players underwent an uncommon offseason, without the normal access to their team’s healthcare providers, strength and conditioning professionals, and high- level coaches. The limited access to these professionals and an absence of the structured preseason preparatory conditioning it was the cause of an unprecedented number of Achilles tendon ruptures in training camp and the beginning of preseason [10]. Unfortunately, these injuries likely represent career-altering and often career-ending events for professional athletes, as one third of the players who sustain an Achilles tendon rupture in the NFL never return back to competition. The remaining two thirds, who are able to return back to play in the NFL following Achilles tendon repair, require approximately 11 months of rehabilitation [11].

It is impossible not to see the great overall risk for elite athletes’ health if a precipitous and not well-planned re-start of the competition worldwide will take place. The risk is greater for team sports where athletes cannot program a single competition, but are instead “forced” into the competitive season with weekly games. Contrary to a classical “return to play” strategy, after this extraordinary training/competition stop, athletes can’t program individual recovery and a slow return to normal athletic condition; thus, a sudden start of the sport season may represent a serious risk for athletes. Moreover, we know that completely asymptomatic subjects may display viral loads similar to those of symptomatic patients; this raises technical questions about the testing not only of all athletes, but also all staff members. How can we avoid the spread of the virus through asymptomatic carriers in teams?

It is important that individual athletes’ physicians, team physicians and sport physicians raise this question with sport federations, warning about a too-rapid resumption of competitions lead by economic interests. As for other society sectors, the COVID-19 lockdown represents economic damage, the classical “black swan” [12] for, in this case, the sport industry. What sport industries’ management should do is to, as other sectors did, prioritize athletes’ health. Many team physicians are now worried about a future increase in injuries among athletes if a correct, long training preparation is not planned before a new start of the competitions. The risk is higher not only for athletes’ health but also for all of the sport industry, considering the long time required for a complete recovery after a muscle–skeletal injury in an elite athlete.

The suggestion is that all the sports federations worldwide, the International Olympic Committee and the sport medicine scientific societies take an unequivocal stand on this topic to protect the health and the careers of athletes of any sport, in any nation.

## Figures and Tables

**Table 1:** Skeletal muscle–tendon–cartilage changes after long term detraining.

Skeletal muscle cross sectional area	decreased
Electromyographic activity	decreased
Muscle strength	decreased
Muscle power	decreased
Tendon tissue structure	impaired
Cartilage lubrification	decreased
